# The association between obesity-related indicators and uncontrolled diabetes mellitus in the elderly: a community-based study in China

**DOI:** 10.3389/fmed.2025.1640888

**Published:** 2025-09-24

**Authors:** Xinxi Zhang, Mingqi Liu, Hangjiong Xuan, Haiping Fang, Xiaojing Yang, Jianqiang Fan

**Affiliations:** ^1^Shaoxing City Shangyu District Center for Disease Control and Prevention, Shaoxing, China; ^2^Shaoxing Center for Disease Control and Prevention, Shaoxing, China

**Keywords:** obesity-related indicators, uncontrolled diabetes mellitus, elderly diabetics, risk thresholds, gender-specific

## Abstract

**Aims:**

Poorly controlled diabetes is closely associated with obesity. This study aimed to investigate the associations and predictive values of obesity-related indicators including waist circumference (WC), body mass index (BMI), weight-adjusted waist index (WWI), and waist-to-height ratio (WHTR) with uncontrolled diabetes mellitus (DM) in an elderly Chinese community population.

**Methods:**

A cross-sectional study was conducted among 15,673 elderly diabetic patients from health examinations in Shangyu District in 2024. Receiver operating characteristic (ROC) curve analysis was used to compare the predictive performance of four obesity indicators for uncontrolled DM. Multivariable logistic regression was performed to assess their associations, while the threshold effects were detected by two piecewise linear models. Subgroup analyses were also performed.

**Results:**

ROC analysis revealed WC had the highest Area under curve (AUC) (0.53, 95%CI: 0.52–0.54), significantly outperforming BMI (*p* < 0.001). Multivariable analysis demonstrated that WC, WWI and WHTR exhibited piecewise linear relationships with uncontrolled DM. Notably, WC showed a nonlinear association only in women (threshold = 70.5 cm), beyond which the risk of uncontrolled DM significantly increased (OR = 1.02, 95%CI: 1.01–1.03). WWI displayed a nonlinear pattern exclusively in men (threshold = 9.60 cm/√kg), with higher values associated with elevated risk (OR = 1.37, 95%CI: 1.24–1.51). WHTR exhibited nonlinear relationships in both genders (thresholds: men = 0.46, women = 0.45), with high risk of uncontrolled DM observed at above thresholds.

**Conclusion:**

WC was the strongest predictor of uncontrolled DM in the elderly population. The nonlinear relationships between WC/WWI and the risk of uncontrolled DM exhibit gender-specific threshold effects.

## 1 Introduction

The global prevalence of diabetes mellitus (DM) is experiencing an unprecedented surge. According to the latest International Diabetes Federation (IDF) epidemiological data, approximately 600 million adults worldwide were living with diabetes in 2024, with projections indicating this number will exceed 850 million by 2050 (1). China, undergoing one of the most rapid demographic transitions globally, demonstrates particularly high diabetes prevalence among its elderly population (2). Notably, despite widespread pharmacological intervention, over 60% of treated patients fail to achieve glycemic control targets (3). Uncontrolled DM not only represents a critical risk factor for microvascular complications, cardiovascular events, and all-cause mortality in elderly populations (4), but also imposes substantial socioeconomic burdens on healthcare systems (5). Therefore, identifying modifiable risk factors and developing targeted intervention strategies for achieving optimal glycemic control are of paramount importance.

Obesity, as a key driver of diabetes mellitus, significantly influences the predictive efficacy of glycemic management through the selection of appropriate assessment metrics. However, the clinical utility of conventional indicators like body mass index (BMI) is increasingly limited in elderly populations (6), as it fails to identify sarcopenic obesity (characterized by concomitant low muscle mass and adiposity) and demonstrates inadequate sensitivity in detecting visceral fat accumulation, which is more closely associated with metabolic risks (7). Numerous studies have showed that central obesity indices such as waist circumference (WC) and waist-to-height ratio (WHTR) exhibit stronger correlations with insulin resistance and glycated hemoglobin (HbA1c) levels (8, 9), consistent with their direct reflection of visceral adipose deposition pathophysiology. The weight-adjusted waist index (WWI), as a novel obesity metric, demonstrates unique theoretical advantages in diabetes risk prediction by dynamically integrating weight and waist circumference changes (10). Accumulating evidence confirms WWI’s robust predictive performance for diabetes risk both in Asian populations and European cohorts (11, 12). However, current findings primarily derive from middle-aged populations, leaving a critical evidence gap regarding its applicability to elderly individuals, particularly those with uncontrolled diabetes mellitus.

Based on the above, the present study systematically evaluates the associations between four obesity-related indicators (BMI, WC, WHTR, and WWI) and uncontrolled DM risk in elderly Chinese community-dwelling populations. We aim to identify the optimal predictive indicator and further elucidate the specific relationships between these obesity indicators and uncontrolled DM risk, thus optimizing glycemic management and intervention strategies in elderly diabetic patients.

## 2 Research design and methods

### 2.1 Research population

**Figure 1:**
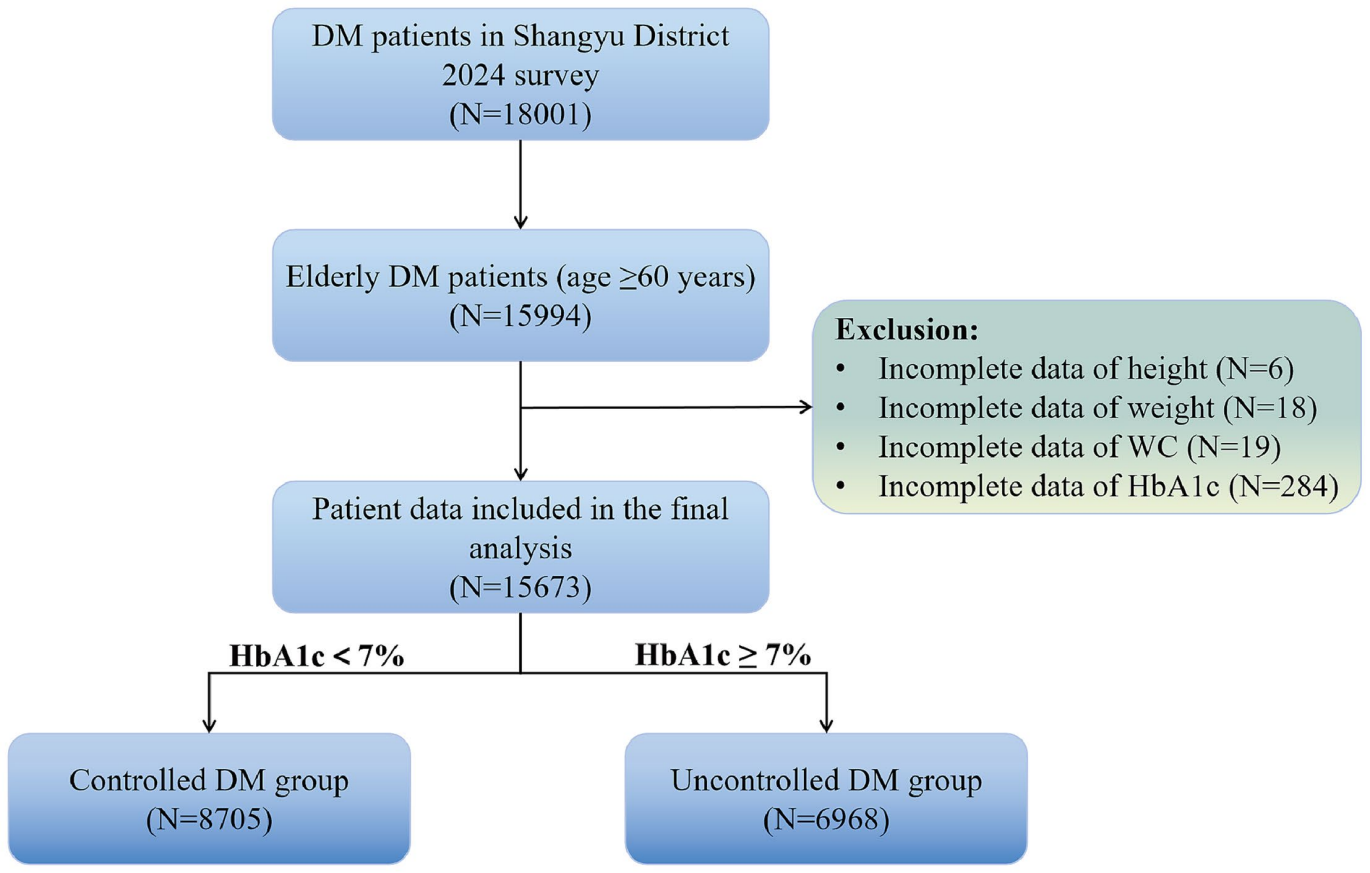
Participant enrollment flowchart.

This cross-sectional study enrolled 15,994 elderly diabetic patients (age ≥60 years) who participated in routine health examinations in Shangyu District, Shaoxing City during 2024. We excluded participants with the missing data for key variables: height (*N* = 6), weight (*N* = 18), WC (*N* = 19), and HbA1c (*N* = 284). The final analytical cohort comprised 15,673 eligible participants. The study protocol received ethical approval (No. SYJK-2025-003). The participant selection process is detailed in Figure 1.

### 2.2 Exposure and outcome

All anthropometric measurements were performed by trained medical staff following standardized protocols. WC was measured at the umbilical level at the end of normal expiration using a flexible tape. BMI was calculated as weight (kg) divided by height squared (m^2^). The WWI was derived using the formula: WC (cm)/√weight (kg). WHTR was computed as WC divided by height. These indicators were analyzed both as continuous variables and as tertile categories (Tertile 1–Tertile 3). Uncontrolled diabetes was defined as HbA1c ≥ 7.0% (13, 14), with all other cases classified as the controlled group.

### 2.3 Covariates

All covariates in this study were collected through standardized health examination questionnaires and physiological measurements. Demographic variables including age and sex were obtained from self-reported questionnaires. Smoking status categorized as current, former, or never smoker. Alcohol consumption frequency classified as daily, 4–6 times per week, 1–3 times per week, or never. Regular exercise habits defined as daily exercise, occasional exercise, or no exercise. Blood pressure in clinical indicators was determined by taking the mean of two resting position measurements. Hypertension was defined as meeting any one of the following criteria: previous diagnosis of hypertension; systolic blood pressure (SBP) ≥ 140 mmHg or diastolic blood pressure (DBP) ≥ 90 mmHg. Dyslipidemia was defined by meeting any of the following criteria (15): total cholesterol (TC) ≥ 5.2 mmol/L (200 mg/dL), low-density lipoprotein cholesterol (LDL-C) ≥ 3.4 mmol/L (130 mg/dL), triglycerides (TG) ≥ 1.7 mmol/L (150 mg/dL), or high-density lipoprotein cholesterol (HDL-C) < 1.0 mmol/L (40 mg/dL) for men and <1.3 mmol/L (50 mg/dL) for women. All data underwent rigorous quality control through double-entry verification and logical consistency checks to ensure accuracy.

### 2.4 Statistical analysis

All statistical analyses were performed using SPSS 26.0 and EmpowerStats (version 4.1). Continuous variables were presented as mean ± standard deviation and categorical variables were expressed as frequencies (percentages). Receiver operating characteristic (ROC) curve analysis was employed to evaluate the predictive performance of each obesity indicator, calculating the area under the curve (AUC), optimal cutoff values, and corresponding sensitivity and specificity. To examine the associations between obesity indicators and risk of uncontrolled DM, we constructed three multivariable logistic regression models: Model 1 (unadjusted), Model 2 (adjusted for age, sex, smoking status, alcohol drinking, and having regular exercises), and Model 3 (adjusted for age, sex, smoking status, alcohol drinking, having regular exercises, SBP, TG, LDL-C, HDL-C, FPG, hypertension, and dyslipidemia). Potential nonlinear relationships were explored using generalized additive models (GAM) and piecewise linear regression, with threshold effects determined by likelihood ratio tests (*p* < 0.05 considered statistically significant). Subgroup analyses were conducted to evaluate potential effect modifications through interaction tests. All statistical tests were two-tailed, with *p* < 0.05 considered statistically significant.

## 3 Results

### 3.1 Baseline characteristics of participants

The study included 15,673 elderly diabetic patients, comprising 6,968 cases with uncontrolled DM and 8,705 controls with controlled DM, as depicted in Table 1. The uncontrolled DM group demonstrated higher proportions of male participants, current smokers, and physically inactive individuals. Obesity-related indicators were consistently elevated in the uncontrolled DM group, including WC, BMI, WWI, and WHTR. Furthermore, the uncontrolled DM group exhibited significantly worse glycemic and lipid metabolic levels (*p* < 0.001).

**Table 1:** Baseline characteristics of the elderly diabetes participants by diabetes control status.

Variables	Elderly patients with controlled DM (*N* = 8,705)	Elderly patients with uncontrolled DM (*N* = 6,968)	*p*-value
Age (years)	71.76 ± 6.56	71.62 ± 6.61	0.235
Male	3,152 (36.21%)	2,708 (38.86%)	<0.001
Smoking status			<0.001
Current	1,071 (12.30%)	1,031 (14.80%)	
Past	182 (2.09%)	155 (2.22%)	
Never	7,452 (85.61%)	5,782 (82.98%)	
Alcohol drinking			<0.001
Everyday	1854 (21.30%)	1,263 (18.13%)	
4–6 times per week	439 (5.04%)	318 (4.56%)	
1–3 times per week	525 (6.03%)	399 (5.73%)	
Never	5,887 (67.63%)	4,988 (71.58%)	
Having regular exercises			<0.001
Regular exercises	2,320 (26.65%)	1,552 (22.27%)	
Less than exercises	1,213 (13.93%)	998 (14.32%)	
No exercise	5,172 (59.41%)	4,418 (63.40%)	
Height (m)	1.59 ± 0.08	1.59 ± 0.08	0.058
Weight (kg)	61.39 ± 10.25	62.05 ± 10.41	<0.001
BMI (kg/m^2^)	24.32 ± 3.30	24.50 ± 3.36	<0.001
Waist circumference (cm)	84.44 ± 8.91	85.40 ± 9.17	<0.001
WWI (cm/√kg)	10.82 ± 0.78	10.88 ± 0.81	<0.001
WHTR	0.53 ± 0.06	0.54 ± 0.06	<0.001
FPG (mmol/L)	6.60 ± 1.27	8.77 ± 2.77	<0.001
HbA1c (%)	6.24 ± 0.47	8.28 ± 1.29	<0.001
SBP (mmHg)	144.03 ± 17.15	145.09 ± 17.48	<0.001
DBP (mmHg)	78.82 ± 9.45	79.73 ± 9.68	<0.001
TC (mmol/L)	4.61 ± 1.09	4.73 ± 1.15	<0.001
TG (mmol/L)	1.56 ± 1.07	1.70 ± 1.24	<0.001
HDL-C (mmol/L)	1.39 ± 0.38	1.37 ± 0.36	0.020
LDL-C (mmol/L)	2.56 ± 0.83	2.68 ± 0.88	<0.001
Hypertension	5,305 (60.94%)	4,423 (63.48%)	0.001
Dyslipidemia	4,603 (52.88%)	4,093 (58.74%)	<0.001

Data are present as *n* (%) or the mean ± standard deviation. BMI, body mass index; WWI, weight-adjusted waist index, WHTR, waist-to-height ratio; FPG, fasting plasma glucose; HbA1c, glycated hemoglobin; SBP, systolic blood pressure; DBP, diastolic blood pressure; TC, total cholesterol; TG, triglyceride; HDL-C, high-density lipoprotein cholesterol; HDL-C, low-density lipoprotein cholesterol.

### 3.2 Obesity indicators predicting uncontrolled DM in elderly

**Figure 2:**
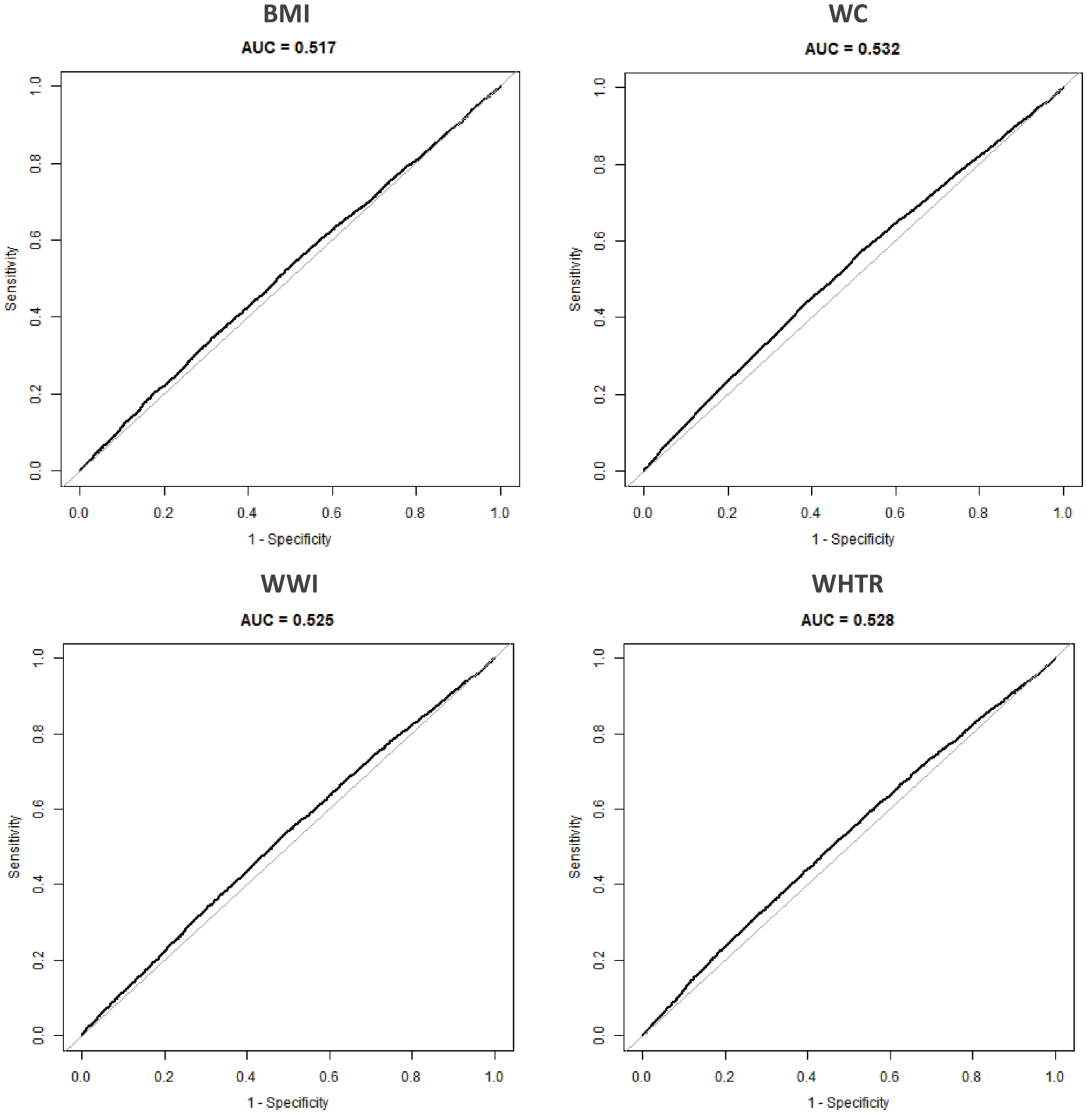
Comparison of the predictive value of the four obesity-related indicators for uncontrolled DM. WC, waist circumference; BMI, body mass index; WWI, weight-adjusted waist index, WHTR, waist-to-height ratio; AUC, area under curve.

The predictive performance of four obesity indicators for uncontrolled DM was evaluated through ROC curve analysis (Figure 2, Table 2). WC demonstrated the highest discriminative ability (AUC = 0.53, 95%CI: 0.52–0.54), with an optimal cutoff of 83.75 cm, at which point the best sensitivity was 57% sensitivity and specificity was 48%. WC-derived indicators, WHTR and WWI, showed comparable but slightly inferior predictive utility (AUC = 0.528 and 0.525, respectively), with no statistically significant differences compared to WC (*p* = 0.090 and *p* = 0.095). In contrast, BMI exhibited significantly inferior predictive value (AUC = 0.517, 95%CI: 0.516–0.534; *p* < 0.001 compared to WC), these findings supported the exclusion of BMI from subsequent analyses, focusing instead on WC, WHTR, and WWI to investigate their associations with uncontrolled DM risk.

**Table 2:** Comparison of AUC values among four obesity-related indicators.

Test	AUC	95% CI	Best threshold	Specificity	Sensitivity	*p* for different in AUC
WC	0.53	0.52, 0.54	83.75	0.48	0.57	Reference
BMI	0.52	0.51, 0.53	23.97	0.49	0.54	<0.001
WWI	0.52	0.52, 0.53	10.78	0.50	0.54	0.095
WHTR	0.53	0.52, 0.54	0.54	0.55	0.49	0.090

AUC, area under the curve; 95% CI, 95% confidence interval.

### 3.3 The association between obesity indicators and uncontrolled DM

Progressive adjustment in multivariable regression models revealed increasingly stronger positive associations between WC, WWI, and WHTR with uncontrolled DM risk (Table 3). As continuous variables, each 1 cm increment in WC was associated with a 1.02-fold higher uncontrolled DM risk (OR = 1.02, 95% CI: 1.01–1.02) in Model 3, while tertile-based categorization demonstrated a 35% elevated risk in the highest tertile compared to the lowest (OR = 1.35, 95% CI: 1.22–1.48). Notably, WWI and WHTR exhibited similar dose–response relationships with uncontrolled DM risk in fully adjusted models. For WWI, each unit increase was associated with an 19% increase in uncontrolled DM risk, while the highest tertile showed an OR of 1.44 (95% CI: 1.30–1.59) versus the lowest tertile; for WHTR, the continuous variable OR reached 14.45 (95% CI: 7.35–28.44) and the highest tertile OR was 1.43 (95% CI: 1.30–1.58). These findings demonstrate robust associations between central obesity indicators and uncontrolled diabetes risk across different analytical approaches.

**Table 3:** Association between central obesity indicators (WC, WWI, WHTR) and uncontrolled DM in elderly patients.

Variables	Model 1		Model 2		Model 3	
	OR (95%CI)	*p*-value	OR (95%CI)	*p*-value	OR (95%CI)	*p*-value
WC (continuous variable)	1.01 (1.01, 1.015)	<0.001	1.01 (1.01, 1.02)	<0.001	1.02 (1.01, 1.02)	<0.001
WC (categorical variable)						
Tertile 1	Reference		Reference		Reference	
Tertile 2	1.08 (0.99, 1.17)	0.069	1.08 (0.99, 1.17)	0.058	1.12 (1.01, 1.23)	0.027
Tertile 3	1.27 (1.17, 1.38)	<0.001	1.27 (1.17, 1.38)	<0.001	1.35 (1.22, 1.48)	<0.001
WWI (continuous variable)	1.11 (1.07, 1.15)	<0.001	1.16 (1.11, 1.21)	<0.001	1.19 (1.13, 1.26)	<0.001
WWI (categorical variable)						
Tertile 1	Reference		Reference		Reference	
Tertile 2	1.13 (1.05, 1.22)	0.002	1.17 (1.09, 1.27)	<0.001	1.28 (1.16, 1.40)	<0.001
Tertile 3	1.23 (1.14, 1.33)	<0.001	1.32 (1.22, 1.44)	<0.001	1.44 (1.30, 1.56)	<0.001
WHTR (continuous variable)	4.56 (2.66, 7.82)	<0.001	6.29 (3.59, 11.01)	<0.001	14.45 (7.35, 28.44)	<0.001
WHTR (categorical variable)						
Tertile 1	Reference		Reference		Reference	
Tertile 2	1.11 (1.03, 1.20)	0.008	1.14 (1.05, 1.23)	0.001	1.22 (1.11, 1.34)	<0.001
Tertile 3	1.25 (1.16, 1.35)	<0.001	1.30 (1.20, 1.41)	<0.001	1.43 (1.30, 1.58)	<0.001

OR, Odd ratio; 95% CI, 95% confidence interval.

Model 1, No covariates were adjusted.

Model 2, Adjusted for age, sex, smoking status, alcohol drinking and having regular exercises.

Model 3, Adjusted for age, sex, smoking status, alcohol drinking, having regular exercises, SBP, TG, LDL-C, HDL-C, FPG, hypertension and dyslipidemia.

### 3.4 Nonlinear association between obesity indicators and uncontrolled DM

**Figure 3:**
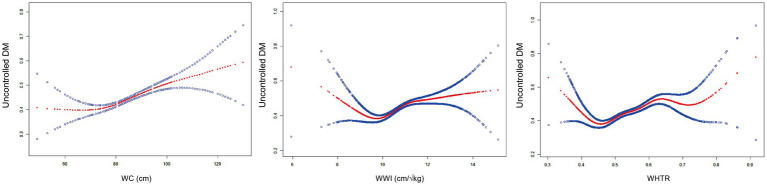
Nonlinear relationship between central obesity indicators (WC, WWI, WHTR) and uncontrolled DM. All analyses were adjusted for age, sex, smoking status, alcohol drinking, having regular exercises, SBP, TG, LDL-C, HDL-C, FPG, hypertension and dyslipidemia.

Nonlinear Association analysis showed significant threshold effects between WC, WWI, WHTR and uncontrolled diabetes (Figure 3; Table 4). A piecewise linear regression model found that the inflection point of WC was 70.5 cm, which had no significant effect on the risk of uncontrolled DM when WC was below this threshold, and when WC exceeded this threshold, the risk of uncontrolled DM was significantly increased (OR = 1.02,95% CI: 1.01–1.02). WWI presented an inflection point at 9.62 cm/√kg, with the low-threshold segment being inversely associated with the risk of uncontrolled DM (OR = 0.63,95% CI: 0.43–0.92) and the high-threshold segment having a sharp increase in risk (OR = 1.23,95% CI: 1.17–1.30). The threshold effect of WHTR was the most significant (*k* = 0.45), and the OR of the low threshold segment tended to be 0 (*p* = 0.004) due to small sample size, while the risk of uncontrolled DM in the high threshold segment surged to 22.81 times (*p* < 0.001).

**Table 4:** Threshold effect analysis of central obesity indicators (WC, WWI, WHTR) on uncontrolled DM in elderly patients using a two-piecewise linear regression model.

Variables	WC	WWI	WHTR
Fitting by standard linear model			
OR (95%CI)	1.02 (1.01, 1.02)	1.19 (1.13, 1.26)	14.45 (7.35, 28.44)
*p*-value	<0.001	<0.001	<0.001
Fitting by two-piecewise linear model			
Breakpoint (K)	70.50	9.62	0.45
OR1 (<K)	0.97 (0.93, 1.01) 0.096	0.63 (0.43, 0.92) 0.016	0.00 (0.00, 0.06) 0.004
OR2 (≥K)	1.02 (1.01, 1.02) < 0.001	1.23 (1.17, 1.30) < 0.001	22.81 (11.13, 46.77) < 0.001
Likelihood ratio test *p*-value	0.015	<0.001	<0.001

Adjusted for age, sex, smoking status, alcohol drinking, having regular exercises, SBP, TG, LDL-C, HDL-C, FPG, hypertension and dyslipidemia.

**Figure 4:**
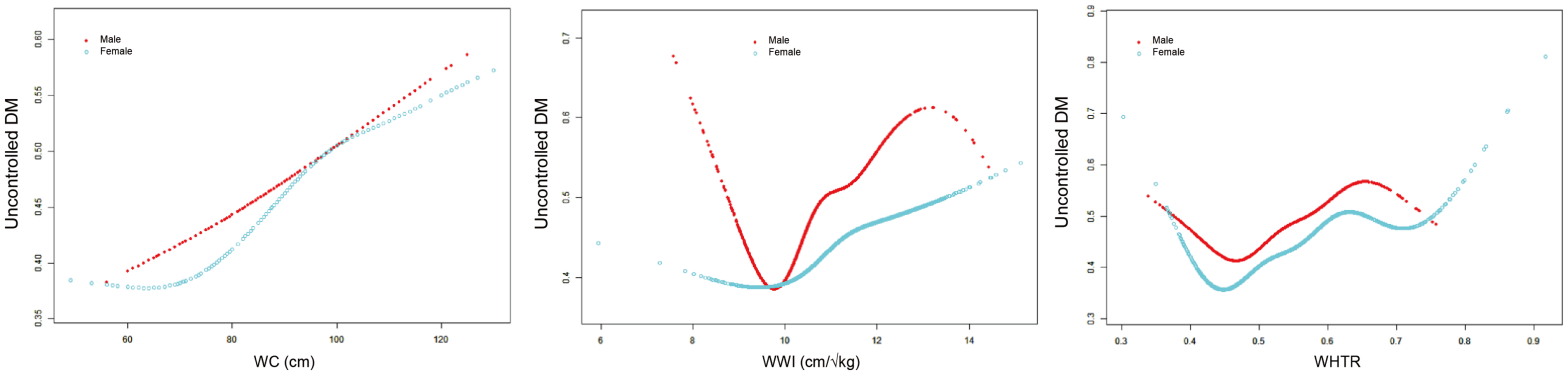
Gender-stratified analyses revealed distinct nonlinear relationships between central obesity indicators (WC, WWI, WHTR) and uncontrolled DM. All analyses were adjusted for age, smoking status, alcohol drinking, having regular exercises, SBP, TG, LDL-C, HDL-C, FPG, hypertension and dyslipidemia.

As these indicators are closely related to gender, further gender-stratified analysis revealed differential threshold effects (Figure 4, Table 5). A significant threshold effect between WC and uncontrolled DM was found only in women, with an inflection point of 70.5 cm as before. In contrast, a significant threshold effect (*k* = 9.60 cm/√kg) between WWI and uncontrolled DM was found only in men, with the same trend above. The threshold effect between WHTR and uncontrolled DM was not affected by sex. These findings suggest a sex-specific metabolic mechanism for the effect of WC and WWI on uncontrolled DM risk.

**Table 5:** Sex-specific threshold effect analysis of central obesity indicators (WC, WWI, WHTR) on uncontrolled DM in elderly patients using a two-piecewise linear regression model.

Variables	WC		WWI		WHTR	
	Male	Female	Male	Female	Male	Female
Fitting by standard linear model						
OR (95%CI)	1.01 (1.01, 1.02)	1.02 (1.01, 1.02)	1.26 (1.15, 1.38)	1.16 (1.09, 1.24)	12.04 (3.60, 40.26)	16.17 (7.01, 36.84)
*p*-value	0.001	<0.001	<0.001	<0.001	<0.001	<0.001
Fitting by two-piecewise linear model						
Breakpoint (K)	78.00	70.50	9.60	10.02	0.46	0.45
OR1 (<K)	0.98 (0.96, 1.01) 0.281	0.97 (0.93, 1.01) 0.171	0.48 (0.29, 0.81) 0.060	0.88 (0.63, 1.23) 0.452	0.00 (0.00, 1.56) 0.068	0.00 (0.00, 1.19) 0.055
OR2 (≥K)	1.02 (1.01, 1.03) < 0.001	1.02 (1.01, 1.03) < 0.001	1.37 (1.24, 1.51) < 0.001	1.19 (1.11, 1.28) < 0.001	28.91 (7.31, 114.38) < 0.001	24.04 (10.03, 57.63) < 0.001
Likelihood ratio test *p*-value	0.054	<0.001	<0.001	0.101	0.009	0.008

Adjusted for age, smoking status, alcohol drinking, having regular exercises, SBP, TG, LDL-C, HDL-C, FPG, hypertension and dyslipidemia.

### 3.5 Subgroup analysis

**Figure 5:**
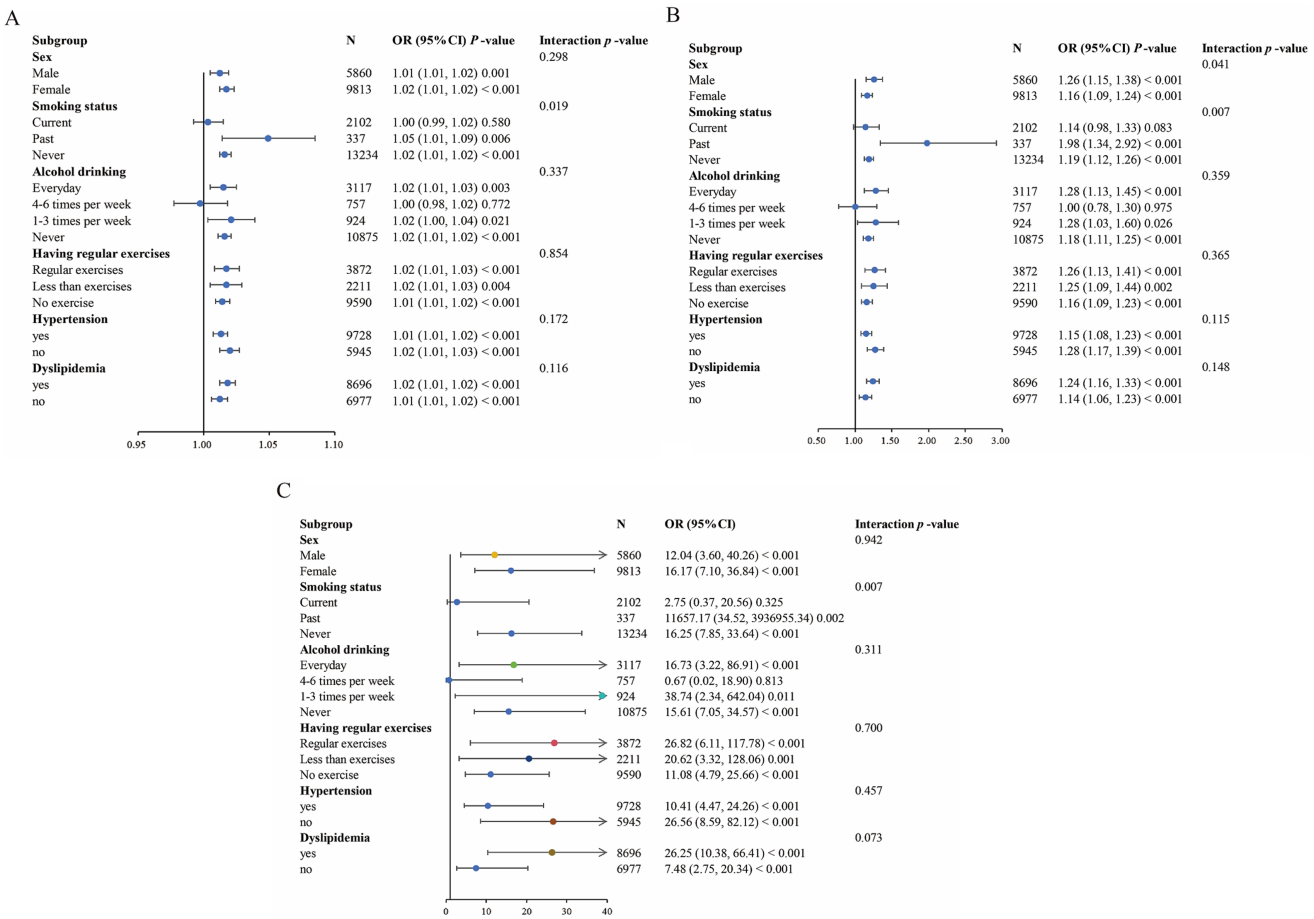
Forest plot of the association between central obesity indicators and uncontrolled DM. **(A)** Forest plot of WC. **(B)** Forest plot of WWI. **(C)** Forest plot of WHTR. Each subgroup analysis adjusted for age, smoking status, alcohol drinking, having regular exercises, SBP, TG, LDL-C, HDL-C, FPG, hypertension and dyslipidemia, except the subgrouping variables.

The results of subgroup analysis are shown in Figure 5. The risk effects of WWI and uncontrolled DM were significantly different between sexes (*p* for interactions <0.05). Additionally, the risk effects of WC, WWI, and WHTR on uncontrolled DM were significantly moderated by smoking status (*p* for interactions <0.05). No other significant interaction was observed in subgroup analyses. Above results suggest that the relationships between uncontrolled DM and these three indices are generally stable in different populations.

## 4 Discussion

Our cross-sectional study revealed complex relationships between obesity indicators and glycaemic control in elderly DM Chinese patients. Among the assessment metrics, WC demonstrated superior predictive performance for uncontrolled DM, significantly outperforming traditional BMI. The WC-derived indicators, WWI and WHTR, showed intermediate predictive values. Dose–response analyses confirmed significant positive associations between all central obesity indicators and uncontrolled DM risk, while nonlinear modeling identified striking gender-specific threshold effects. WC exhibited a threshold effect exclusively in women (inflection point = 70.5 cm), whereas WWI showed male-specific threshold behavior (inflection point = 9.60 cm/√kg), beyond which uncontrolled DM risk increased substantially. These findings provide critical insights for risk stratification in elderly diabetic populations.

The inferior predictive performance of BMI can be explained through multiple mechanisms. From a body composition perspective, the prevalent sarcopenic obesity in elderly populations (with 1–2% annual muscle loss concurrent with fat accumulation) renders BMI inadequate for assessing true metabolic risk (16, 17). In contrast, WC, WHTR and WWI demonstrate superior ability to quantify visceral adipose tissue (VAT) deposition, which represents the primary pathophysiological driver of metabolic dysregulation (18). VAT might impact glucose metabolism through: (1) enhanced lipolytic activity increasing portal free fatty acid flux; (2) disordered adipokine production, particularly involving leptin and resistin, promotes insulin resistance; and (3) macrophage-mediated chronic low-grade inflammation impairing insulin signaling (19–21). These mechanisms collectively explain the superior diagnostic utility of central obesity measures. However, the above obesity-related indicators did not show sufficient AUC values (0.52–0.53), which may be related to the following reasons: (1) aging-related metabolic heterogeneity, where the prevalent sarcopenic obesity disrupts fat-muscle proportion equilibrium (22), thereby attenuating the linear correlation between conventional anthropometric measures and glycemic control; (2) the multisystem influences on HbA1c, which reflects not only adiposity but also insulin resistance, β-cell function, and red blood cell lifespan, making it broader than obesity indicators; and (3) sex-aging interactions, where postmenopausal estrogen decline in women drives adipose redistribution and age-related androgen reduction in men exacerbates muscle loss-both processes potentially compromising the predictive efficacy of single indicators for glycemic outcomes (23).

Gender-specific threshold effects of WC/WWI for uncontrolled DM constituted another key finding in our study. The WC threshold only found in women (70.5 cm vs. conventional 80 cm cutoff for obesity) likely reflects postmenopausal metabolic alterations. The significant decline in estrogen levels among elderly women influences adipose tissue distribution through multiple pathways: attenuated suppression of adipocyte differentiation promotes preadipocyte maturation, elevated cortisol accumulation in adipose tissue exacerbates visceral obesity, and shifted macrophage polarization toward proinflammatory phenotypes occurs in fat depots (24, 25). Collectively, these alterations contribute to aberrant fat distribution and subsequent metabolic dysregulation (26). It is worth noting that a China-based study also demonstrated that increase of WC was specifically associated with elevated risks of adverse metabolic phenotypes exclusively in elderly female populations (27). Conversely, the male-specific WWI threshold (9.597 cm/√kg) may reflect unique body composition characteristics, including higher lean mass and distinct fat distribution patterns in male participants. WWI standardizes waist circumference by body weight, thereby enhancing its ability to reflect the muscle-to-fat mass ratio, thereby offering enhanced precision in assessing systemic metabolic status (28, 29). When WWI exceeds the threshold, it may indicate that males have entered a state of metabolically obese normal weight (MONW) (30). The underlying mechanisms likely involve androgen decline-induced muscle loss with concomitant visceral fat accumulation, coupled with elevated β3-adrenergic receptor expression in male adipose tissue that potentiates lipolysis (31, 32), thereby establishing a male-specific pathophysiological cascade that exacerbates metabolic dysregulation and diabetes progression. WHTR showed strong associations with the uncontrolled DM risk in both genders but higher ORs in men, consistent with their greater propensity for central adiposity and more severe metabolic derangements (33, 34).

Our study carries important clinical relevance in two key aspects. First, this study provides the first comprehensive comparison of four obesity indicators (BMI, WC, WWI, WHTR) for predicting uncontrolled DM in elderly populations, addressing a critical evidence gap. Second, the identification of gender-specific thresholds (70.5 cm WC in women; 9.60 cm/√kg WWI in men) reveals important sexual dimorphism in obesity-related metabolic risk. Certainly, several limitations should be considered when interpreting our findings. The cross-sectional design precludes causal inference, necessitating validation in prospective cohorts. In addition, the regional focus on Eastern China in participant recruitment could reduce external validity, necessitating future studies with multiethnic cohorts.

## 5 Conclusion

In conclusion, WC and WWI emerge as optimal predictors of poor glycemic control, exhibiting gender-specific threshold patterns in elderly diabetics. Higher risks of uncontrolled DM could be observed in elderly female diabetic patients with WC ≥ 70.5 cm or in elderly male diabetic patients with WWI ≥ 9.60 cm/√kg, which may have important clinical implications for personalizing glucose-lowering strategies in the growing population of older adults with diabetes.

## Data Availability

The raw data supporting the conclusions of this article will be made available by the authors, without undue reservation.
